# Multimodal Imaging Investigation of the Dentato‐Thalamo‐Cortical Pathway in Friedreich's Ataxia

**DOI:** 10.1002/mds.70179

**Published:** 2026-01-21

**Authors:** Yinghua Jing, Imis Dogan, Ravi Dadsena, Jennifer Faber, Jörg B. Schulz, Kathrin Reetz, Sandro Romanzetti, the FACROSS study group, Stella A. Lischewski, Kerstin Konrad, Miguel Pishnamaz, Maximillian Praster, Thomas Clavel, Vera Jankowski, Joachim Jankowski, Oliver Pabst, Katharina Marx‐Schütt, Nikolaus Marx, Julia Möllmann, Malte Jacobsen, Juergen Dukart, Simon Eickhoff, Ralf‐Dieter Hilgers

**Affiliations:** ^1^ Translational Neurodegeneration, Department of Neurology RWTH Aachen University Aachen Germany; ^2^ JARA‐Brain Institute Molecular Neuroscience and Neuroimaging (INM‐11), Research Centre Jülich and RWTH Aachen University Aachen Germany; ^3^ German Center for Neurodegenerative Diseases (DZNE) Bonn Germany; ^4^ Department of Parkinson's Disease Sleep and Movement Disorders, Center for Neurology, University Hospital of Bonn Bonn Germany; ^5^ Department of Neuroradiology University Hospital Bonn Bonn Germany

**Keywords:** dentate nucleus, diffusion tensor imaging, dynamic causal model, phosphorus magnetic resonance spectroscopy, thalamus

## Abstract

**Background:**

Friedreich's ataxia (FRDA) is a spinocerebellar neurodegenerative disorder. The dentato‐thalamo‐cortical (DTC) pathway, an important cerebellar output involved in motor control, plays a crucial role in the neural mechanisms underlying ataxia symptoms in FRDA.

**Objective:**

The aim was to quantify regional alterations in structure, connectivity, function, and neurometabolism along the DTC pathway in FRDA patients using multimodal magnetic resonance imaging (MRI).

**Methods:**

Twenty‐two individuals with FRDA and 22 healthy controls underwent a brain MRI. Volumetry, amplitude of low‐frequency fluctuation of resting‐state functional MRI data, and phosphorus MR spectroscopy were used to assess key regional changes along the DTC pathway. Diffusion tractography and dynamic causal model (DCM) were adopted to investigate microstructural integrity and effective connectivity of the DTC pathway, respectively. Associations with clinical parameters, including ataxia severity, were also tested.

**Results:**

Compared to controls, FRDA patients exhibited reduced volumes and adenosine triphosphate levels in the bilateral dentate nuclei and right motor cortex, as well as elevated glycerophosphoethanolamine levels in thalami and the left motor cortex. In FRDA patients, fractional anisotropy was decreased in the dentatothalamic sections of the DTC tract and correlated negatively with ataxia severity. Additionally, DCM revealed elevated excitatory connectivity from the right thalamus to the left dentate nucleus in FRDA patients, showing a U‐shaped association with ataxia scores.

**Conclusions:**

This study provides multimodal imaging evidence for comprehensive alterations along the DTC pathway in FRDA, including first insights into energy metabolism and effective connectivity. A better pathophysiological understanding of early metabolic and dynamic pathway disruptions might inform potential neuromodulatory interventions targeting this pathway. © 2026 The Author(s). *Movement Disorders* published by Wiley Periodicals LLC on behalf of International Parkinson and Movement Disorder Society.

Friedreich's ataxia (FRDA) is the most common autosomal recessive ataxia, typically characterized by progressive motor incoordination, dysarthria, and cardiomyopathy.^1^, ^2^, ^3^, ^4^ FRDA is mainly caused by the GAA trinucleotide repeat expansions in the first intron of the frataxin gene,^5^, ^6^ with the number of GAA repeats being closely associated with disease onset and progression.^2^ The deterioration of ataxia symptoms could be reliably monitored using the Scale for the Assessment and Rating of Ataxia (SARA).^3^

The dentate nucleus is a key region of neuropathology in FRDA.^7^ Pathological evidence has shown visible atrophy in the gray matter structure and its efferent myelinated fibers on sagittal sections of the cerebellar hemispheres.^7^, ^8^, ^9^, ^10^ Previous longitudinal studies also indicated that degeneration in the dentate nucleus could be recognized in the early disease stage and is linked to ataxia progression.^11^, ^12^ The dentate nucleus receives information from the cerebellar cortex and connects through the contralateral thalamus to the contralateral cerebral cortex such as the motor cortex, forming the dentato‐thalamo‐cortical (DTC) pathway.^13^ This pathway is a vital cerebellar efferent pathway and is well recognized for involvement in motor control, including timing, initiation, coordination, and execution.^14^, ^15^, ^16^ Thus, investigation of key brain regions and connectivity along the DTC pathway in FRDA might provide promising biomarkers for monitoring disease progression and evaluating therapeutic outcomes, as well as provide insights into the development of treatment strategies targeting this pathway to slow ataxia progression.

Magnetic resonance imaging (MRI) provides noninvasive approaches to characterize the structural, functional, and neurometabolic profiles along the DTC pathway. Particularly, voxel‐based morphometry (VBM) is an automated method that assesses regional volumes,^17^ and diffusion tractography helps the visualization and detection of the DTC tract microstructure integrity.^18^, ^19^, ^20^ Amplitude of low‐frequency fluctuation (ALFF) measures the local intensity of spontaneous blood‐oxygen‐level‐dependent signals, reflecting intrinsic neuronal activity.^21^ Additionally, the dynamic causal model (DCM) can explicitly model the directed functional interactions between the dentate nucleus, thalamus, and motor cortex, incorporating neural mass models that account for synaptic responses and neuronal population dynamics.^22^, ^23^, ^24^ Phosphorus magnetic resonance spectroscopy (^31^P‐MRS) offers the opportunity to directly detect energy and phospholipid metabolites in vivo.^25^ These approaches are widely applied in various neurodegenerative disease research.^26^, ^27^, ^28^, ^29^, ^30^ However, brain ^31^P‐MRS and DCM have, to the best of our knowledge, not been applied in FRDA so far.

Previous tractography studies have noted DTC tract alterations in FRDA.^31^, ^32^, ^33^, ^34^ More recently, Cocozza and colleagues specifically mapped the gradient of microstructural damage of the DTC tract in FRDA patients, indicating anterograde degeneration from the dentate nucleus toward the motor cortex.^34^ However, these studies mainly evaluated the white matter integrity of the DTC tract and do not capture function or neurometabolism. To address this gap, we designed a multimodal MRI study to quantify regional changes (volumetric, functional activity, and energy metabolism) in the dentate nucleus, thalamus, and motor cortex, as well as network‐level alterations (microstructural integrity and effective connectivity) of the DTC pathway in FRDA. Therefore, this study aims to provide a comprehensive understanding of the DTC‐pathway involvement in FRDA and provides new insights for further research into potential monitoring and therapeutic options (eg, metabolism and neuromodulatory interventions) targeting this pathway.

## Patients and Methods

This study was conducted as an MRI substudy as part of the European Friedreich's Ataxia Consortium for Translational Studies (EFACTS)^1^, ^2^, ^3^ at the University Hospital RWTH Aachen, Germany, and approved by the local ethics committee (EK083/15 and EK057/10, RWTH Aachen University, Germany). All participants provided written informed consent.

### Participants

Twenty‐two patients with genetically confirmed FRDA and 22 healthy controls matched for age, sex, and handedness were enrolled in this cross‐sectional study. Demographic and clinical information, including SARA to quantify ataxia severity, is summarized in Table 1.

**TABLE 1:** Participant demographics and clinical information

	FRDA (N = 22)	Control (N = 22)	Between‐group difference (*p*‐value)
Demographics			
Sex (female/male)	11/11	12/10	0.763
Handedness (R/L/M)	19/2/1	20/2/0	0.599
Age (yr)	36.14 ± 12.62	37.09 ± 11.51	0.794
Clinical data			
Age of onset (yr)	16.23 ± 7.61	–	–
Disease duration (yr)	19.77 ± 9.34	–	–
GAA1	540.55 ± 210.64	–	–
GAA2	867.95 ± 189.65	–	–
SARA	17.57 ± 7.39	–	–
ADL	13.68 ± 5.44	–	–

*Note*: Data are reported as mean ± standard deviation or number of subjects.

Abbreviations: FRDA, Friedreich's ataxia; N, number of subjects; R, right; L, left; M, mixed; GAA1, GAA repeat length on the smaller FXN allele; GAA2, GAA repeat length on the larger FXN allele; SARA, Scale for the Assessment and Rating of Ataxia; ADL, Activities of Daily Living.

### MR Procedures

^1^H‐MRI measurements, including T1‐weighted structural imaging, resting‐state functional MRI (rs‐fMRI), and diffusion‐weighted imaging, as well as ^31^P‐MRS measurements, were obtained on a 3‐T PRISMA scanner (Siemens Healthineers, Erlangen, Germany). Details of the methods and parameters of MR acquisition are provided in Supporting Information S1.

### Region‐of‐Interest Identification

Anatomical masks of the bilateral dentate nuclei, thalami, and motor cortices were defined as six key regions of interest (ROI) in the DTC pathway. The dentate nuclei masks from a spatially unbiased atlas template (spatially unbiased infratentorial template [SUIT])^35^ were used for subsequent imaging analyses, except for tractography analysis. Masks of the thalamus and precentral gyrus obtained from the Mori Atlas^36^ were used to define thalamic and motor cortex ROIs.

For tractography analysis, the dentate seed ROIs were derived from an in‐house developed deep neural network^37^ in subject‐specific space to facilitate accurate reconstruction of the DTC. Additionally, to improve the specificity of fiber tracking and reduce spurious streamlines in the guided tractography, superior cerebellar peduncles (SCP) were used as waypoint regions, and red nuclei were included as loose spatial constraints, with masks for these regions from the Mori Atlas.^36^

### Volumetric Analysis

The volume of the dentate region was estimated using the SUIT atlas‐based segmentation approach (https://www.diedrichsenlab.org/imaging/suit.htm). Briefly, it included cerebellum and brainstem isolation, manual correction, normalization to the SUIT template, and Jacobian modulation.^19^ Volumes of the thalamus and motor cortex regions were derived from VBM analysis using the *CAT12* toolbox (https://neuro-jena.github.io/cat//) with default parameter settings. Processing steps included tissue segmentation, spatial normalization to the Montreal Neurological Institute (MNI) space, modulation, and total intracranial volume (TIV) estimation.^38^ All volumes were normalized as a percentage of TIV.

### ALFF Analysis

After rs‐fMRI preprocessing (Supporting Information S2.1), 1 FRDA patient was excluded due to head movement (translations in any direction >3.0 mm or rotation >3°). ALFF was calculated using *RESTPlus* (version *1.24*)^39^ as follows: (1) removal of the linear trend in time course; (2) noise factor regression of head movement parameters (using Friston 24),^40^ cerebrospinal fluid (CSF), and white matter; (3) time course conversion of the signal in each voxel to the power spectrum by a fast Fourier transform; (4) square‐root averaging of the power amplitude in 0.01 to 0.08 Hz; and (5) normalization to the global ALFF mean. Finally, the mean ALFF value in each ROI was extracted for each subject.

### Phosphate Metabolite Analysis

An integrative processing pipeline combining structural MRI and ^31^P‐MRS data was implemented for cross‐modal spatial metabolite analysis. Briefly, raw spectroscopic data were preprocessed and fitted in *TARQUIN* (version *4.3.11*).^41^ All spectral fitting results passed quality assessment and visual inspection. Then, we used scripts based on *R* (version *4.4.1*) and the *ANTsR* package (version *0.6.1*)^42^ to derive metabolic spatial maps of the following eight metabolites for each subject: phosphocreatine (PCr), inorganic phosphate (Pi), adenosine triphosphate (ATP), nicotinamide adenine dinucleotide (NAD(H)), phosphoethanolamine (PE), phosphocholine (PCh), glycerophosphoethanolamine (GPE), and glycerophosphocholine (GPC). Next, T1‐weighted structural images were segmented and normalized to MNI spaces. Subsequently, each ^31^P metabolite image was resampled into MNI space using b‐spline interpolation via T1‐weighted structural images. We also obtained the estimated contribution of tissue types within each original ^31^P‐MRS voxel in the MNI space. After partial volume correction for CSF, the signal amplitude of each metabolite was extracted for each ROI and was expressed as a percentage of the total phosphorus signals in the corresponding spectrum. See Supporting Information S2.2 for details.

### Dentato‐Thalamo‐Cortical Tract Reconstruction

All ROIs were transformed into each subject's diffusion space. After preprocessing (Supporting Information S2.3), bilateral tractography was conducted using the *MRtrix3* (version *3.0.4*)^43^ toolbox using the “tckgen” command in combination with the “iFOD2” algorithm,^44^ utilizing white matter fiber orientation distribution maps as input. Briefly, the DTC tract reconstruction was generated from the dentate nucleus to the contralateral motor cortex target, with the constraint that streamlines traversed the ipsilateral SCP, contralateral red nucleus, and contralateral thalamus. To maximize anatomical coverage, this process was repeated bidirectionally (from the dentate nucleus to the motor cortex and vice versa). The resulting streamlines were filtered to remove anatomically implausible reconstructions by constraining streamline length to the median length of each bundle. Subsequently, the whole tract was converted into a mask using the “tckedit” command and was subdivided into five distinct sections: from the dentate nucleus to the SCP, the SCP, from the SCP through the red nucleus to the thalamus, the thalamus, and from the thalamus to the motor cortex. Each whole‐tract mask and individual sections were then used to extract relevant diffusion metrics of fractional anisotropy (FA), mean diffusivity (MD), and radial diffusivity (RD) from their corresponding parameter maps for subsequent analysis.

### DCM Analysis

The effective connectivity of the DTC pathway was estimated using a deterministic two‐state DCM^24^ in *SPM12* (https://www.fil.ion.ucl.ac.uk/spm/). After preprocessing (Supporting Information S2.1), rs‐fMRI data were modeled using a general linear model (GLM), accounting for six head motion parameters, white matter, and CSF signals as nuisance regressors. Then, the principal eigenvariate time series of six key ROIs was extracted from the GLM of each participant to represent average regional activity. A connectivity model based on anatomical priors, including six self‐connections of ROIs and eight interconnections between the dentate nucleus and the thalamus as well as between the thalamus and the motor cortex, was developed to assess the comprehensive and interactive influences of the DTC pathway (Fig. S1). Subsequently, the parametric empirical Bayes^45^, ^46^ framework was applied to model individual‐ and group‐level effects. At the individual level, subject‐wise parameters were estimated and inverted to find the estimation of DCMs, including connection strength and the corresponding model evidence. At the group level, a design matrix was created for group average (commonality) and between‐group comparisons (FRDA patients vs. controls), applying empirical shrinkage priors. Finally, a Bayesian model average was calculated over possible models from the final iteration of the greedy search, weighted by the posterior probability (PP) of models.^46^

### Statistical Analysis

Statistical analyses were performed using *IBM SPSS Statistics 24* and R (version *4.4.1*). Normality of distribution and heterogeneity of variances were checked using the Shapiro–Wilk test and Levene's test, respectively. Age difference between FRDA patients and controls was tested using two‐sample *t* tests, and categorical variables (sex and handedness) were tested using Pearson's χ^2^ tests. A GLM with age and gender as covariates was used to compare differences in regional volume, ALFF, and metabolite between groups. *p* < 0.05 was considered statistically significant.

Due to the different diffusion tensor imaging data acquisitions, two‐way analysis of variance (ANOVA) and Eta squared (*η* ^2^) were conducted to assess the effects of group and acquisition sequence on each diffusion metric of the DTC tract. *η* ^2^ values of 0.01, 0.06, and 0.14 were considered as small, medium, and large effect sizes, respectively.^47^ For diffusion metrics not influenced by sequence (*p* > 0.05, *η* ^2^ < 0.14), two‐sample *t* tests were subsequently applied to compare group differences for the whole tract and individual sections along the DTC tract; for diffusion metrics significantly affected by sequence (*p* < 0.05, *η* ^2^ ≥ 0.14), no between‐group comparison was performed. The significance threshold was set at *p* < 0.05. Additionally, Bayesian statistics were adopted to estimate the posterior distributions of DCM parameters using *SPM12*. Rather than the *p*‐value in traditional statistical significance testing, PP > 0.8 was considered clear evidence for a nonspurious effect (real effective connectivity).^48^, ^49^

To further investigate relationships with disease severity in FRDA patients, Pearson's correlation analyses between diffusion parameters of the DTC tract and clinical scores were performed for the whole DTC tract and individual sections, separately. As two‐state DCM incorporates excitatory and inhibitory connectivity, its relationship with clinical scores may not be simply linear. Therefore, we additionally applied quadratic regression analysis to capture nonlinear relationships with altered effective connectivity. As our main focus was on DTC‐pathway alterations in relation to ataxia severity, we report correlation analyses using SARA, whereas associations of imaging metrics with other clinical measures are detailed in the Supporting Information. As an exploratory analysis, significance was set at *p* < 0.05 without correction for multiple comparisons.

## Results

### Volume and Spontaneous Neural Activity

Volumes of the bilateral dentate regions and the right motor cortex were significantly decreased in FRDA patients compared to controls (Table 2). No significant group differences were found in the ALFF values.

**TABLE 2:** Comparisons of volume and ALFF in key brain regions of the DTC pathway between FRDA patients and controls

	FRDA (N = 22)	95% CI	Control (N = 22)	95% CI	Between‐group difference (*p‐*value)
Volume (%)					
Left dentate nucleus	0.09 ± 0.01	[0.09, 0.10]	0.10 ± 0.01	[0.10, 0.11]	**<0.001**
Right dentate nucleus	0.10 ± 0.01	[0.10, 0.11]	0.11 ± 0.01	[0.11, 0.12]	**<0.001**
Left thalamus	0.26 ± 0.03	[0.25, 0.28]	0.26 ± 0.04	[0.24, 0.28]	0.436
Right thalamus	0.25 ± 0.03	[0.24, 0.27]	0.25 ± 0.04	[0.23, 0.26]	0.423
Left motor cortex	1.00 ± 0.11	[0.95, 1.04]	1.04 ± 0.11	[0.99, 1.09]	0.099
Right motor cortex	0.82 ± 0.09	[0.78, 0.86]	0.86 ± 0.09	[0.82, 0.90]	**0.037**
ALFF					
Left dentate nucleus	0.40 ± 0.12	[0.35, 0.45]	0.43 ± 0.12	[0.37, 0.48]	0.453
Right dentate nucleus	0.40 ± 0.12	[0.35, 0.46]	0.41 ± 0.12	[0.35, 0.46]	0.965
Left thalamus	0.49 ± 0.07	[0.46, 0.53]	0.50 ± 0.07	[0.47, 0.53]	0.847
Right thalamus	0.46 ± 0.06	[0.43, 0.49]	0.48 ± 0.06	[0.45, 0.51]	0.256
Left motor cortex	1.00 ± 0.10	[0.95, 1.04]	1.00 ± 0.20	[0.91, 1.09]	0.956
Right motor cortex	1.08 ± 0.15	[1.01, 1.15]	1.05 ± 0.17	[0.97, 1.13]	0.568

*Note*: Data are reported as mean ± standard deviation and 95% confidence interval. Bold values indicate statistically significant results (*p* < 0.05). For ALFF, 1 FRDA patient was excluded due to head movement.

Abbreviations: ALFF, amplitude of low‐frequency fluctuation; DTC, dentato‐thalamo‐cortical; FRDA, Friedreich's ataxia; N, number of subjects; %, percentage of total intracranial volume; 95% CI, 95% confidence interval.

### Neural Energy Phosphate Metabolites

**FIG. 1:**
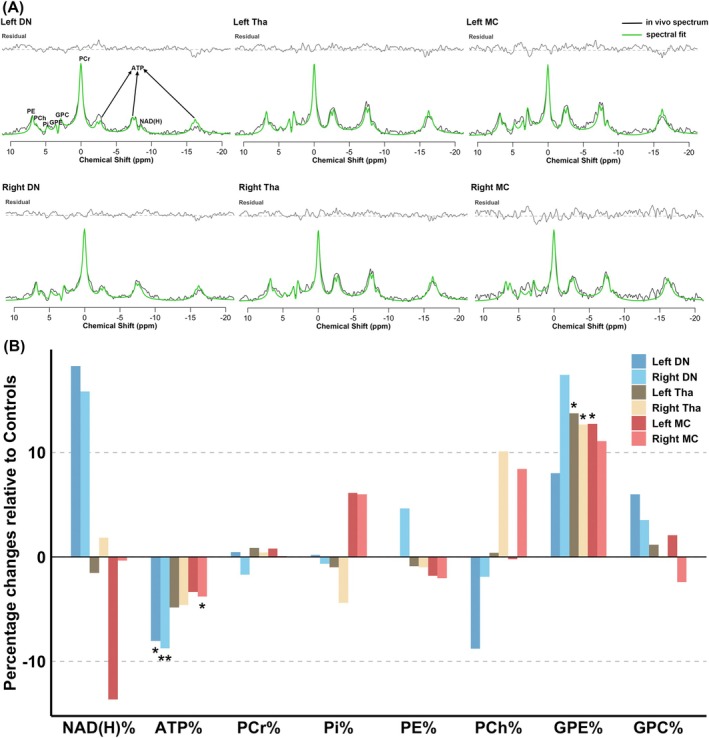
Energy phosphate and phospholipid membrane metabolism profile in key brain regions along the dentato‐thalamo‐cortical pathway. (**A**) Representative spectra and fits from the dentate region, thalamus, and motor cortex of 1 Friedreich's ataxia (FRDA) patient (10‐Hz line broadening for visualization). (**B**) Percentage changes in phosphate metabolites in the dentate region, thalamus, and motor cortex in FRDA relative to controls (*p < 0.05 and **p < 0.01). Percentage difference = 100 × (mean_patient_ – mean_control_)/mean_control_. ATP, adenosine triphosphate; DN, dentate nucleus; GPC, glycerophosphocholine; GPE, glycerophosphoethanolamine; MC, motor cortex; NAD(H), nicotinamide adenine dinucleotide; PCh, phosphocholine; PCr, phosphocreatine; PE, phosphoethanolamine; Pi, inorganic phosphate; Tha, thalamus; %, percentage of the total phosphorous signal. [Color figure can be viewed at wileyonlinelibrary.com]

The ATP levels were lower in FRDA patients relative to controls in bilateral dentate regions (left: −8.02%, *p* = 0.024; right: −8.76%, *p* = 0.010) and the right motor cortex (−3.79%, *p* = 0.048; Fig. 1B). Compared to controls, FRDA patients exhibited higher GPE levels in bilateral thalami (left: 13.74%, *p* = 0.029; right: 12.66%, *p* = 0.030) and the left motor cortex (12.72%, *p* = 0.036; Fig. 1B). Detailed comparisons are presented in Table S1 and Figure S2. Additionally, the ATP level in the left dentate region negatively correlated with SARA (r = −0.44, *p* = 0.040), and the GPE level in the left motor cortex positively correlated with the age of onset (r = 0.46, *p* = 0.031; Fig. S3).

### Microstructural Integrity of Dentato‐Thalamo‐Cortical Tract

Two‐way ANOVA showed significant effects of group on the mean FA of each whole DTC tract (left: F_(1, 41)_=23.27, *p* < 0.001, *η* ^2^ = 0.36; right: F_(1, 41)_=19.69, *p* < 0.001, *η* ^2^ = 0.32), and no influence from acquisition sequence (left: F_(1, 41)_=1.49, *p* = 0.230, *η* ^2^ = 0.03; right: F_(1, 41)_=1.06, *p* = 0.309, *η* ^2^ = 0.03). However, the mean MD and RD of the DTC tract were influenced by both group and sequence (all *p* < 0.001, *η* ^2^ > 0.14), with sequences having larger effects (Table S2). Therefore, only FA was used for subsequent between‐group analysis to reflect microstructural changes.

**FIG. 2:**
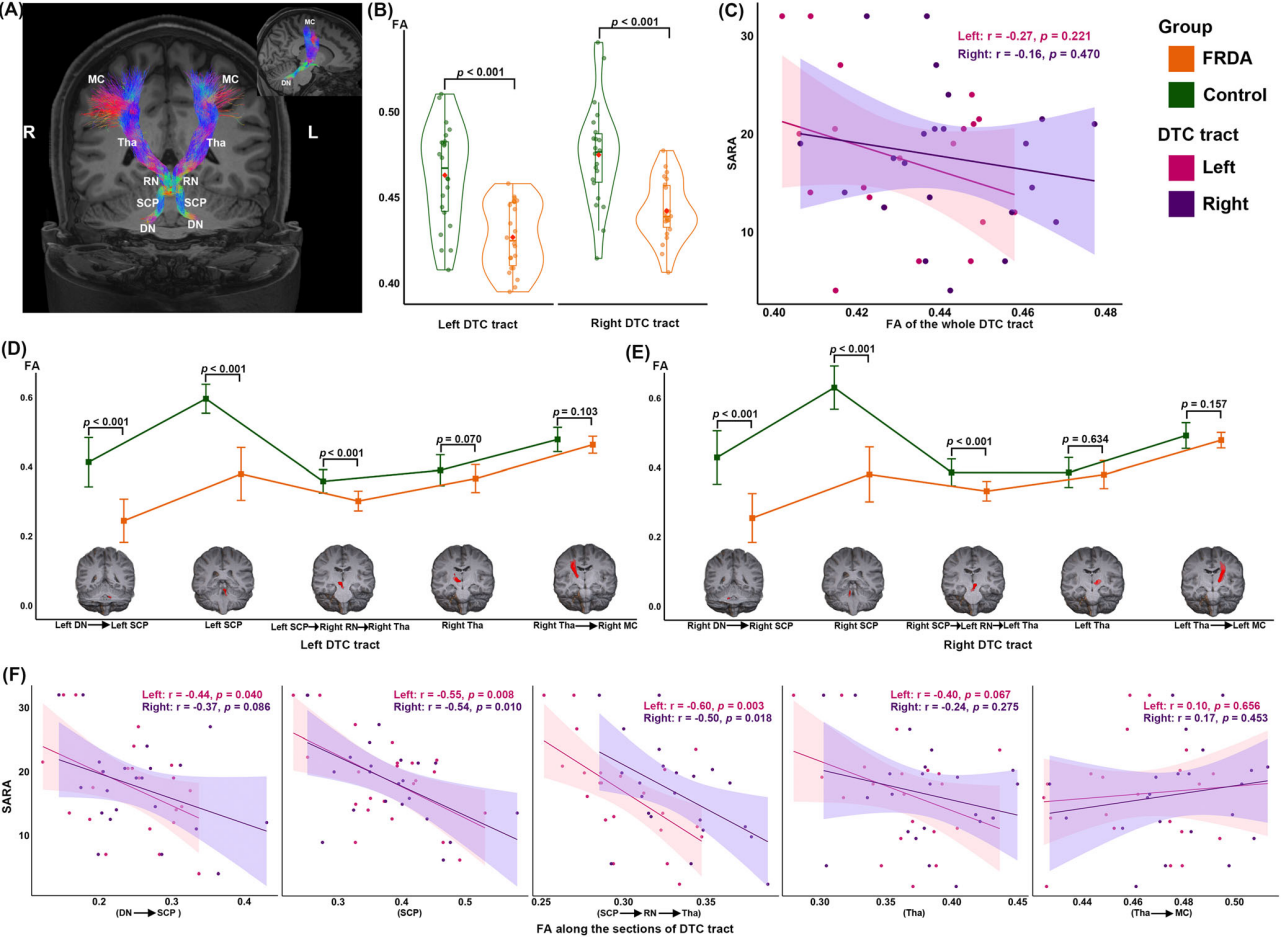
Microstructural changes in the dentato‐thalamo‐cortical (DTC) tract. (**A**) White matter fiber tractography of the bilateral DTC tracts in 1 control subject. (**B**) Group comparisons for mean FA of the bilateral DTC tract. (**C**) Correlation between SARA scores and mean FA of the bilateral DTC tract. (**D**, **E**) Group comparisons for average FA along five sections of the DTC tract. Sections of the DTC tract are presented on the *x*‐axis. (**F**) Correlation between SARA total scores and average FA along each section of the DTC tract. DN, dentate nucleus; DTC, dentato‐thalamo‐cortical; FA, fractional anisotropy; FRDA, Friedreich's ataxia; L, left; MC, motor cortex; R, right; r, Pearson's correlation coefficient; RN, red nucleus; SARA, Scale for the Assessment and Rating of Ataxia; SCP, superior cerebellar peduncles; Tha, thalamus. [Color figure can be viewed at wileyonlinelibrary.com]

Group comparison revealed reduced mean FA of bilateral DTC tract in FRDA patients compared to controls (*p* < 0.001, respectively), but no significant correlations with any clinical scores, including SARA (Fig. 2C; Fig. S4). Regarding average FA along sections of the tract, FRDA patients exhibited pronounced reductions compared to controls in sections from the dentate nuclei to the SCP, bilateral SCP, and from the SCP to the thalami (all *p* < 0.001). In addition, FA along these sections from the dentate nucleus to their contralateral thalami (except for the section from the right dentate nucleus to right SCP) negatively correlated with SARA (Fig. 2F; Table S3). There were no significant correlations with other disease‐related parameters; only Activities of Daily Living (ADL) negatively correlated with the average FA of bilateral SCPs to their contralateral thalami (Fig. S5; Table S3).

### Effective Functional Connectivity of Dentato‐Thalamo‐Cortical Pathway

**FIG. 3:**
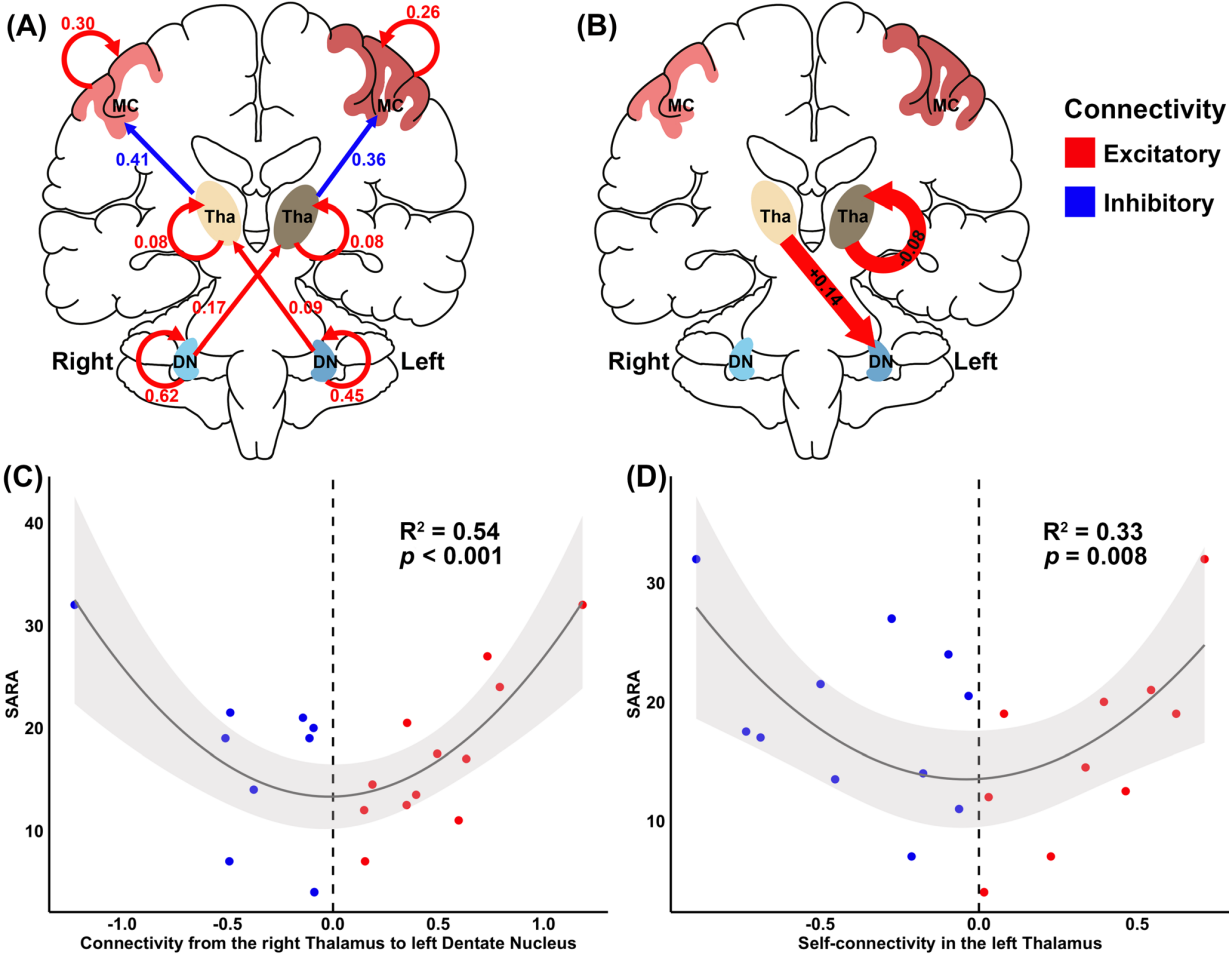
Pattern changes in effective connectivity of the dentato‐thalamo‐cortical (DTC) pathway. (**A**) A schematic of commonality connectivity for the DTC pathway after Bayesian model averaging, with effect sizes for each parameter in the model (posterior probability > 0.8). (**B**) Significant differences in effective connectivity of the DTC pathway between Friedreich's ataxia (FRDA) patients and controls (posterior probability > 0.8). (**C**, **D**) U‐shaped relationship between SARA and each altered connection in FRDA patients. DN, dentate nucleus; MC, motor cortex; R^2^, coefficient of determination; SARA, Scale for the Assessment and Rating of Ataxia; Tha, thalamus. [Color figure can be viewed at wileyonlinelibrary.com]

Across all patients and controls, parameters of within‐group effect included the excitatory connections from the bilateral dentate nuclei to their respective contralateral thalami and inhibitory connections from the bilateral thalami to their respective ipsilateral motor cortices. In addition, the self‐excitatory connection was found in each dentate nucleus, thalamus, and motor cortex (Fig. 3A; Table S4).

Compared to controls, the increased excitatory influence from the right thalamus to the left dentate nucleus (PP = 0.95) and the decreased self‐excitatory influence within the left thalamus (PP = 0.82) were the best discriminative parameters in FRDA patients. Furthermore, significant U‐shaped relationships were found between SARA scores and the two connections in FRDA patients, respectively (Fig. 3C,D). Quadratic associations between all effective functional connections with other clinical parameters are shown in Figure S6.

## Discussion

This multimodal MR imaging study comprehensively characterizes DTC‐pathway alterations in FRDA patients compared to controls, reporting for the first time altered profiles of energy metabolism and effective functional connectivity. Across key regions along the DTC pathway, atrophy and decreased ATP levels were found in the bilateral dentate nuclei and right motor cortex in FRDA patients, as well as increased GPE levels in bilateral thalami and the left motor cortex. Regarding connectivity of the DTC pathway, the damaged microstructural integrity of the DTC tract in FRDA patients correlated clinically with ataxia severity, particularly from the dentate nuclei to the contralateral thalami. Additionally, the effective connectivity pattern of the DTC pathway was altered in FRDA patients, correlating with ataxia severity in a U‐shaped relationship.

Neuropathological evidence in FRDA shows substantial atrophy and cell death of the large glutamatergic neurons in the dentate nucleus.^8^, ^9^ Consistent with previous MRI findings,^10^, ^11^, ^12^ the dentate nucleus, as the origin structure of the DTC pathway, was more significantly impacted than other key pathway regions in FRDA. Particularly, in addition to atrophy, lower ATP levels and an elevated trend of the NAD(H) levels were observed in the bilateral dentate regions in FRDA patients compared to controls. This indicated that the mitochondrial oxidative phosphorylation process in the dentate nuclei was impaired, and the cells were in an energy‐depleted condition.^50^, ^51^ Meanwhile, the elevated NAD(H) level reflects the NAD^+^/NADH cycle disturbance, indicating impaired electron transport chain activity, which further exacerbates the lack of ATP energy supply.^50^, ^52^ Moreover, phospholipid degradation products (GPE and GPC) increased, whereas phospholipid synthesis precursor (PCh) decreased in the dentate nucleus. Although phospholipid membrane metabolism changes did not reach statistical significance, these changes may reflect imbalanced phospholipid membrane turnover, presuming impaired cell membrane reparation and myelin damage in the dentate nucleus in the early stage of FRDA.^51^ In contrast, in the bilateral thalami and left motor cortex, we found only enhanced breakdown of cell membranes. However, we found no significant differences in ALFF, thus assuming that FRDA patients still retain some compensatory functional capacity at this stage. Further exploration of neurometabolic and functional dynamics in longitudinal studies is important to elucidate developmental dysfunction,^53^ as well as to clarify the pathological evolution of compensation and decompensation in FRDA progression. This would optimize stage‐specific metabolic intervention strategies. For example, liposomal NAD^+^ supplementation^54^ or cytidine diphosphate‐ethanolamine^55^ might help to maintain mitochondrial energy and phospholipid metabolic homeostasis in the early stage of FRDA, whereas mitochondrial‐targeting bioenergetics or high‐dose antioxidant compounds such as coenzyme Q10^56^ may be more appropriate for reducing oxidative stress–induced membrane phospholipid degradation to improve motor symptoms during the more severe stage of FRDA.

Regarding microstructural connectivity, the finding of impaired microstructural integrity of the DTC tract is in line with the recent diffusion MRI study.^34^ Unlike their methodology, which used the ComBat harmonization algorithm to harmonize for differences in data acquisition,^34^ we first assessed potential influence factors using ANOVA and Eta squared and then adopted FA metrics that were not influenced by sequence for subsequent analysis. Although we sampled only the DTC tract into five sections rather than 100 equidistant points for profilometry analysis, the pattern of our results closely replicates the findings of Cocozza and colleagues^34^ that microstructural impairments in the DTC tract are weighted to early dentatothalamic sections, with damage severity diminishing along thalamocortical connections. This spatial pattern supports the current hypothesis of an anterograde impairment in FRDA.^8^, ^57^ We also observed negative correlations between FA values and the clinical SARA score in dentatothalamic sections. Particularly, FA along the section from the left SCP to the right thalamus could remain significantly correlated with SARA and ADL after Bonferroni correction, corroborating previous MRI findings and indicating a strong association of microstructural degeneration with motor incoordination in FRDA.^32^, ^33^, ^58^, ^59^

Moreover, we observed significant changes in thalamic effective functional connections of the DTC pathway. These changes showed U‐shaped nonlinear correlations with SARA scores, indicating a complex course from functional compensation to decompensation of the DTC pathway in FRDA patients. Increased excitatory influence from the right thalamus to the left dentate nucleus may reflect a functional compensatory effort to maximize cerebellar activity. Previous task‐based fMRI studies suggested that the cerebro‐cerebellar loop could maintain motor control via functional reorganization at the early stage of FRDA.^60^, ^61^, ^62^ The thalamus, as a critical integrative hub of functional brain networks,^63^ may try to keep the transmission efficiency of the DTC pathway by enhancing inputs to the dentate nucleus when the dentate is pathologically damaged.^64^ However, it remains to be further investigated in large‐scale longitudinal studies whether this hyperexcitatory connectivity contributes to functional maintenance or is merely a state of pathological hyperexcitability. In addition, decreased self‐excitatory connectivity within the left thalamus may imply its reduced capacity for information integration. With the loss of efficient inputs from the dentate nucleus, synchronization of neuronal activity within the thalamus is reduced, resulting in impaired motor coordination control.^65^ These findings provide new insights to better understand the functional reorganization of the DTC pathway in FRDA patients. Transcranial direct current stimulation (tDCS) has been shown to reduce motor and cognitive symptoms in FRDA patients.^66^ Due to the importance and plasticity of the DTC pathway in FRDA, noninvasive neuromodulation targeting this pathway, such as tDCS and transcranial magnetic stimulation (TMS), might promote compensatory mechanisms in the early stage of FRDA and may slow ataxia progression. Thus, it is worthwhile to investigate the dynamic changes in effective connectivity of the DTC pathway during disease progression to obtain an in‐depth pathophysiological understanding of developmental and degeneration changes as well as develop personalized and precise noninvasive therapeutic strategies.

### Limitations and Outlook

Several additional limitations and potential areas for further work should be considered. First, this study was a cross‐sectional design with a small sample (only adult FRDA patients in less‐advanced stages), preventing the inference of progression changes or subgroup differences in the DTC pathway. Although we characterized the relationship between disease severity and altered connectivity of the DTC pathway, we must be cautious in extending these exploratory outcomes clearly to underlying biological mechanisms, and it is essential to carry out large‐scale longitudinal studies to understand the DTC‐pathway involvement in FRDA progression. Second, metabolite levels were normalized to total ^31^P; absolute quantification would be more informative. Additionally, ^31^P‐MRS has a low spatial resolution, and its partial volume effects remain unavoidable, especially in the dentate region. As shown in Figure S7, metabolism signals extracted using masks could be influenced by the signals from white matter or other cerebellar structures. Furthermore, we noted ATP fitting imperfections in some voxels, reflecting *TARQUIN*'s weakness in simulating non‐Lorentzian in vivo metabolite spectra lineshapes and linewidths. Despite potentially affecting fitting precision, these imperfections did not change the main conclusions. Thus, future ^31^P‐MRS studies at 7‐T MRI or with single‐voxel MRS using alternative fitting approaches like LCModel are needed to validate our findings. Finally, DCM analysis was based on rs‐fMRI. Future exploration could be conducted by combining the DTC‐dependent tasks (eg, finger tapping and oculomotor tasks) or noninvasive brain stimulation techniques (eg, tDCS and TMS) to reveal the DTC pathway changes during specific motor control in FRDA patients, which would also be valuable for therapeutic evaluation.

## Conclusion

This study provides multimodal imaging evidence for regional and connectivity changes along the DTC pathway in FRDA patients, especially from the dentate nuclei to the thalami. Our structural atrophy, energy deficiency, and damaged integrity of the DTC tract support the hypothesis that pathological change in the DTC pathway begins in the dentate nuclei and progressively involves the motor cortices. Besides, the altered pattern of effective connectivity indicates that the thalamus plays an important regulatory role in the functional plasticity of the DTC pathway. Together, our work provides new insights into developing and evaluating FRDA treatment strategies targeting the DTC pathway.

## Author Roles

(1) Research project: A. Conception and design, B. Organization, C. Execution; (2) Statistical analysis: A. Design, B. Execution, C. Review and critique; (3) Manuscript: A. Writing of the first draft, B. Review and critique.

Y.J.: 1A, 1B, 1C, 2A, 2B, 3A

I.D.: 1A, 1B, 2C, 3B

R.D.: 2C, 3B

J.F.: 2C,3B

J.B.S.: 2C, 3B

K.R.: 1A, 1B, 2C, 3B

S.R.: 1A, 1B, 1C, 2C, 3B

## Financial Disclosures and Conflicts of Interest

Author disclosures are available in the Supporting Information.

## Full financial disclosures of all authors for the preceding 12 months

I.D., S.R., and K.R. are employees of the RWTH Aachen University and were supported by the Interdisciplinary Center for Clinical Research within the Faculty of Medicine at the RWTH Aachen University, Germany (OC2). J.F. was funded within the Advanced Clinician Scientist Programme (ACCENT, funding code 01EO2107, by the German Federal Ministry of Education and Research [BMBF]) and as a principal investigator of the iBehave Network, sponsored by the Ministry of Culture and Science of the State of North Rhine‐Westphalia and received funding from the National Ataxia Foundation (NAF); J.F. has received consultancy honoraria from Vico Therapeutics and Biogen, unrelated to the present study. Otherwise, Y.J., R.D., and J.B.S. have no financial disclosures or conflicts of interest related to this research.

## Supporting information


**Data S1.** Supporting Information

## Data Availability

The data that support the findings of this study are available from the corresponding author upon reasonable request.

## Appendix A Further FACROSS Study Group Members

Stella A. Lischewski (Department of Neurology, University Hospital RWTH Aachen); Kerstin Konrad (Section Child Neuropsychology, Department of Child and Adolescent Psychiatry, Psychosomatics and Psychotherapy, University Hospital, RWTH Aachen); Miguel Pishnamaz, Maximillian Praster (Department for Orthopaedics, Trauma and Reconstructive Surgery, University Hospital RWTH Aachen); Thomas Clavel (Functional Microbiome Research Group, Institute of Medical Microbiology, University Hospital RWTH Aachen); Vera Jankowski, Joachim Jankowski (Institute for Molecular Cardiovascular Research, University Hospital RWTH Aachen); Oliver Pabst (Institute of Molecular Medicine, University Hospital RWTH Aachen); Katharina Marx‐Schütt, Nikolaus Marx, Julia Möllmann, Malte Jacobsen (Department of Internal Medicine I, Cardiology, University Hospital RWTH Aachen); Juergen Dukart, Simon Eickhoff (Institute of Neuroscience and Medicine, Brain and Behaviour [INM‐7], Research Centre Jülich); and Ralf‐Dieter Hilgers (Department of Medical Statistics, University Hospital RWTH Aachen, Germany).
