# Supplementary material for: Multimodal Imaging Investigation of the Dentato‐Thalamo‐Cortical Pathway in Friedreich's Ataxia
Source: Mov Disord. 2026 Jan 21;41(4):909–20. doi: 10.1002/mds.70179 (PMC13067320; doi:10.1002/mds.70179)
Supplement: Supplementary file 1 — Data S1. Supporting Information [file MDS-41-909-s001.zip › Supplementary_Material_MDS70179(MDS-25-1308 ).pdf]

Supplementary Material

1 MR Acquisition 3

1.1 [1]H-MRI measurements 3

1.2 ^{31}P-MRS measurements 4

2 Details on Data Processing 5

2.1 Functional Data Preprocessing 5

2.2 Spectroscopic Data Processing 5

2.3 Diffusion Data Preprocessing 7

TABLE S1. Comparisons of phosphate metabolites in key regions along the DTC pathway between patients with Friedreich's ataxia and controls. 8

TABLE S2. Two-way ANOVA results for the influence of different sequences and groups on mean tract profiles of the whole DTC tract. 11

TABLE S3. Comparison of significant correlations between original Pearson's correlation and partial correlation controlling for age and sex. 12

TABLE S4. Results of the Bayesian dynamic model within-group and between-group. 13

FIGURE S1. Illustration for a dynamic causal model network of the dentato-thalamo-cortical pathway. 15

FIGURE S2. Group comparison of phosphate metabolites in each key brain region between patients with Friedreich's ataxia and controls. 16

FIGURE S3. Correlation map illustrating Pearson's correlation coefficients between clinical scores and all metabolites in each key region along the dentato-thalamo-cortical pathway in Friedreich's ataxia patients 17

FIGURE S4. Scatterplots and linear regression plots between clinical scores and mean FA of the whole dentato-thalamo-cortical tract in Friedreich's ataxia patients. 19

FIGURE S5. Scatterplots and linear regression plots between average FA along each section of the dentato-thalamo-cortical tract and clinical scores in Friedreich's ataxia patients. 20

FIGURE S6. Quadratic regression analyses between effective connections and clinical scores in Friedreich's ataxia patients......21

FIGURE S7. Dentate nucleus selection of ^{31}P-MRS displayed on axial and coronal anatomical images......22

Supplementary References......23

1 MR Acquisition

All brain imaging data were performed on a PRISMA (Siemens Healthineers, Erlangen, Germany) 3T whole-body scanner. Proton magnetic resonance imaging (^{1}H-MRI) measurements were conducted on a whole-body transmitter coil with a 64-channel head-neck coil. Phosphorus magnetic resonance spectroscopy (^{31}P-MRS) measurements were performed using a Dual-tuned transmit and receive birdcage head coil produced by Rapid Biomed (Rimpar, Germany).

1.1 ^{1}H-MRI measurements

T1-weighted structural images were obtained with a 3D magnetization-prepared rapid gradient echo sequence with 208 sagittal slices, isotropic voxel of 0.80×0.8×0.8 mm, repetition time of 2400 ms, echo time of 2.36 ms, inversion time of 1000 ms, flip angle of 8°, and field of view of 288×288 mm.

Resting-state functional MRI (rs-fMRI) data were collected with a $T2^{*}$-weighted echo-planar imaging sequence with interleaved slice acquisition. Each scan consisted of 205 whole-brain volumes containing 36 axial slices (3.1 mm thickness) with a plane voxel size of 3.1×3.1 mm, repetition time of 2221 ms, echo time of 30 ms, flip angle of 90°, and field of view of 384×384 mm. During rs-fMRI measurement, participants were instructed to close their eyes, remain still, stay awake, and not think of anything in particular. This measurement was performed in a dark environment.

Diffusion-weighted images were performed using a multi-slice spin-echo planar imaging sequence with the following parameters: repetition time of 2680 ms, echo time of 75.6 ms, flip angle of 78°, voxel size of 2×2×2 mm, field of view of 208×208 mm, and 72 slices. The imaging plane was aligned parallel to the AC-PC line, with the phase-encoding in the anterior-posterior direction. The field of view was adjusted to ensure coverage of the brainstem, cerebellum, and cortex. The diffusion encoding protocol consisted of 16 non-diffusion-

weighted reference scans (b=0 s/mm^2) followed by two distinct diffusion weightings: 51 directions each at b=1000 s/mm^2 and b=2000 s/mm^2, with diffusion gradient directions uniformly distributed across the spherical coordinate system. For a few subjects (3 controls and 9 patients), the double-shell diffusion gradient scheme was not available, so a single-shell diffusion-weighted acquisition scheme with a b=1000 s/mm^2 and 67 diffusion gradient directions was performed (repetition time of 6800 ms, echo time of 81 ms, flip angle of 90°, voxel size of 2.4×2.4×2.4 mm, field of view of 216×216 mm, and 64 slices).

1.2 ^{31}P-MRS measurements

To enhance the registration between ^{31}P metabolite images and structural T1-weighted images, low-resolution T1-weighted reference images were acquired with a 3D gradient echo sequence using the coil's proton channel of the dual-tuned coil. A total of 72 frames were obtained with a voxel size of 4×4×4 mm, repetition time of 752 ms, echo time of 7.65 ms, flip angle of 50°, and field of view of 256×224×288 mm.

^{31}P-MRS data were acquired with a whole-brain 3D chemical shift imaging (CSI) sequence. A non-selective square RF pulse (duration 0.7 ms) was used to achieve a flip angle of 40°. The minimum echo time available of 2.3 ms was used, during which aphase-encoding gradient was applied. The acquisition spanned 512 ms, during which WALTZ4 1H decoupling was implemented during the first 256 ms. Repetition time was 2000 ms, field of view was 260×260×260 mm, matrix size was 10×10×10, and acquisition time was 13.3 min. Before acquisition, automatic 3D shimming was executed to optimize the homogeneity of the magnetic field within a rectangular shimming volume, which encompassed the whole brain coverage while constrained by the skull. If necessary, the field homogeneity was further manually adjusted until a value of <25 Hz was obtained for full width at half maximum of the absolute-value ^{1}H MR water signal. K-space was sampled using a weighted elliptical phase-encoding strategy with four averages. The volume of interest was aligned along the midline and parallel

to the AC-PC line. Spatial post-processing involved zero-filling to achieve a matrix size of 16×16×16. The nominal volume of the measured voxels was approximately 17.5 cm^3, which, according to Pohmann and von Kienlin[1], effectively assumes a spherical shape with a volume of approximately 40 cm^3.

2 Details on Data Processing

2.1 Functional Data Preprocessing

All rs-fMRI data were preprocessed using SPM12 (https://www.fil.ion.ucl.ac.uk/spm/) and RESTPlus (version 1.24)[2]. The first 10 time points were discarded to allow the magnetization to reach a steady state. Then, functional images were slice-timing corrected to correct slice-dependent delays. Head motion was corrected by registering raw images to the structural image using six parameters (3 translations and 3 rotations). Successively, spatial normalization to the Montreal Neurological Institute (MNI) space was performed using the deformation fields derived from T1 image unified segmentation (resampling voxel size = 3.0×3.0×3.0 mm). After checking the realignment and normalization results, all images were smoothed with a 6 mm full width at half maximum (FWHM) Gaussian kernel. One patient with Friedreich's ataxia (FRDA) was excluded from further functional analyses due to head movement (translations in any direction > 3.0 mm or rotation > 3°).

2.2 Spectroscopic Data Processing

^{31}P spectra were automatically fitted by TARQUIN (version 4.3.11)[3], which provided voxel-wise amplitudes of ^{31}P metabolites. Before the fitting procedure, ^{31}P-MRS data were pre-processed with filtering, zero-filling, phase correction, baseline correction, and frequency shifting to 0 ppm for phosphocreatine (PCr). Spectra fitting was performed with a simulated ^{31}P brain basis set in the time domain using prior knowledge[4]. TARQUIN's default simulated metabolite basis uses very narrow intrinsic lines (Lorentzian FWHM ≈ 0.8 Hz) at generation time; in fitting, TARQUIN then applies additional global and per-metabolite damping to match

the in vivo linewidth. The following metabolite resonances were simulated using a single Lorentzian peak: PCr (0 ppm), inorganic phosphate (Pi, 4.82 ppm), nicotinamide adenine dinucleotide (NAD(H), -8.3 ppm), phosphoethanolamine (PE, 6.72 ppm), phosphocholine (PCh, 6.24 ppm), glycerophosphoethanolamine (GPE, 3.5 ppm), and glycerophosphocholine (GPC, 2.93 ppm). Adenosine triphosphate (ATP) was simulated using three resonances: γ-ATP (-2.6 ppm) and α-ATP (-7.62 ppm) with doublet peaks and β-ATP (-16.26 ppm) with triplet peaks. Two MR spectroscopists (YJ and SR) assessed the quality of fitting results according to recently published consensus criteria[5].

Subsequently, we used scripts based on R (version 4.4.1) and the ANTsR package (version 0.6.1)[6, 7] to extract and create metabolic spatial maps of the above eight metabolites for each subject. Afterward, to facilitate the extraction of metabolite values from anatomical masks, we resampled each ^{31}P metabolite image to the MNI space using b-spline interpolation via T1-weighted structural images. The processing steps are as follows: (1) T1-weighted structural images segmentation using FastSurfer[8]; (2) individual T1-weighted structural images normalization to the MNI template via symmetric normalization diffeomorphic registration, optimizing cross-correlation similarity through multi-resolution iterations[9]; (3) rigid alignment between low-resolution T1-weighted reference images and T1-weighted structural images; (4) each ^{31}P metabolite image spatially co-registration to the MNI space applying geometric transformations obtained through above two registrations (resampling voxel size = 1.0×1.0×1.0 mm); (5) generating a CSI grid image in ^{31}P space using the deformation fields derived from T1 image unified segmentation for subsequent extraction of tissue partial volumes within each CSI voxel in MNI space. All transformations were quality-controlled through overlay visualization and landmark correlation checks. After partial volume correction for CSF, the total phosphor signal was defined as the sum of all fitted phosphor metabolites' signal

amplitudes in each spectrum, and each metabolite was expressed as a percentage of the total phosphor signal.

2.3 Diffusion Data Preprocessing

Raw diffusion data were visually inspected for brain abnormalities or severe artifacts. Then, images were preprocessed using commands according to MRtrix3 guiding principles with the following steps[10]: (1) denoising using 'dwidenoise'; (2) removal of Gibbs ringing artifacts using 'mrdegibbs'; (3) adjustments for susceptibility-induced and eddy-current-induced distortions, participant motion, and slice dropout artifacts, achieved through 'dwifslpreproc'; (4) bias field correction using 'dwibiascorrect', (5) registration of T1-weighted structural images to the diffusion space; (6) computing diffusion tensors using the 'dwi2tensor' algorithm and generating individual diffusion tensor imaging (DTI) metric maps of fractional anisotropy (FA), mean diffusivity (MD), and radial diffusivity (RD) using 'tensor2metric'.

Before the dentato-thalamo-cortical (DTC) tract reconstruction, we also generated fiber orientation distributions (FODs) using the 'dwi2fod' command from MRtrix3. For multishell diffusion acquisitions, the 'msmt_csd' algorithm was implemented[11], while 'csd' was applied for single-shell diffusion acquisitions[12].

TABLE S1. Comparisons of phosphate metabolites in key regions along the DTC pathway between patients with Friedreich's ataxia and controls.

Key brain region	FRDA (N=22)	Control (N=22)	Between-group difference (*p* value)
**PCr (%)**			
Left Dentate Nucleus	39.73 ± 3.09	39.55 ± 2.29	0.828
Right Dentate Nucleus	38.80 ± 2.39	39.46 ± 2.26	0.354
Left Thalamus	30.75 ± 1.54	30.48 ± 1.49	0.551
Right Thalamus	30.60 ± 1.47	30.47 ± 1.49	0.773
Left Motor Cortex	31.80 ± 2.29	31.55 ± 1.69	0.684
Right Motor Cortex	31.95 ± 2.00	31.92 ± 2.12	0.960
**Pi (%)**			
Left Dentate Nucleus	6.04 ± 1.78	6.02 ± 0.99	0.978
Right Dentate Nucleus	6.00 ± 1.04	6.04 ± 1.08	0.904
Left Thalamus	7.11 ± 0.99	7.18 ± 0.94	0.812
Right Thalamus	6.82 ± 0.96	7.14 ± 0.95	0.291
Left Motor Cortex	7.43 ± 0.98	7.00 ± 1.16	0.201
Right Motor Cortex	7.24 ± 1.31	6.83 ± 0.93	0.220
**ATP (%)**			
Left Dentate Nucleus	16.51 ± 2.30	17.95 ± 1.86	**0.024**
Right Dentate Nucleus	16.85 ± 1.73	18.47 ± 2.42	**0.010**
Left Thalamus	21.54 ± 2.12	22.64 ± 1.56	0.054
Right Thalamus	21.68 ± 2.29	22.72 ± 1.43	0.070
Left Motor Cortex	21.90 ± 1.74	22.65 ± 1.69	0.149
Right Motor Cortex	21.91 ± 1.64	22.78 ± 1.20	**0.048**

**NAD(H) (%)**			
Left Dentate Nucleus	4.80 ± 1.88	4.06 ± 0.93	0.105
Right Dentate Nucleus	4.68 ± 1.94	4.04 ± 1.06	0.161
Left Thalamus	4.35 ± 1.12	4.42 ± 0.71	0.811
Right Thalamus	4.44 ± 1.48	4.36 ± 0.73	0.818
Left Motor Cortex	4.20 ± 1.37	4.87 ± 0.94	0.072
Right Motor Cortex	4.65 ± 1.15	4.67 ± 0.69	0.954
**PCh (%)**			
Left Dentate Nucleus	5.01 ± 1.52	5.50 ± 0.96	0.202
Right Dentate Nucleus	5.12 ± 1.15	5.22 ± 0.95	0.761
Left Thalamus	4.52 ± 0.88	4.50 ± 0.71	0.940
Right Thalamus	4.85 ± 1.00	4.40 ± 0.89	0.137
Left Motor Cortex	4.33 ± 0.85	4.34 ± 0.79	0.972
Right Motor Cortex	4.66 ± 1.66	4.30 ± 0.88	0.376
**PE (%)**			
Left Dentate Nucleus	12.10 ± 2.05	12.10 ± 1.25	0.989
Right Dentate Nucleus	12.46 ± 1.72	11.91 ± 1.02	0.212
Left Thalamus	11.75 ± 1.47	11.86 ± 1.00	0.773
Right Thalamus	11.68 ± 1.86	11.80 ± 1.20	0.797
Left Motor Cortex	11.93 ± 1.40	12.14 ± 1.29	0.545
Right Motor Cortex	11.89 ± 2.16	12.14 ± 1.28	0.612
**GPC (%)**			
Left Dentate Nucleus	10.24 ± 2.38	9.66 ± 1.02	0.290
Right Dentate Nucleus	10.13 ± 1.24	9.78 ± 1.10	0.312
Left Thalamus	12.53 ± 1.51	12.38 ± 1.07	0.705

Right Thalamus	12.52 ± 1.89	12.54 ± 0.83	0.973
Left Motor Cortex	12.08 ± 1.44	11.83 ± 1.30	0.513
Right Motor Cortex	11.59 ± 2.00	11.87 ± 1.33	0.549
**GPE (%)**			
Left Dentate Nucleus	5.57 ± 1.86	5.16 ± 1.46	0.393
Right Dentate Nucleus	5.95 ± 1.63	5.07 ± 1.79	0.082
Left Thalamus	7.45 ± 1.40	6.55 ± 1.26	**0.029**
Right Thalamus	7.41 ± 1.17	6.58 ± 1.29	**0.030**
Left Motor Cortex	6.33 ± 1.35	5.61 ± 0.89	**0.036**
Right Motor Cortex	6.11 ± 1.51	5.50 ± 0.86	0.109

Note: Data are reported as mean ± standard deviation. Bold values indicate statistically significant results ($p < 0.05$).

Abbreviations: DTC, dentato-thalamo-cortical; FRDA, Friedreich's ataxia; N, number of subjects; %, percentage of the total phosphorous signal; PCr, phosphocreatine; Pi, inorganic phosphate; ATP, adenosine triphosphate; NAD(H), nicotinamide adenine dinucleotide; PE, phosphoethanolamine; PCh, phosphocholine; GPE, glycerophosphoethanolamine; GPC, glycerophosphocholine.

TABLE S2. Two-way ANOVA results for the influence of different sequences and groups on mean tract profiles of the whole DTC tract.

**Factor**	**Df**	**F Value**	***p* Value**	**η^2**
**FA (Left DTC tract)**				
Group	1	23.27	**< 0.001**	0.36
Sequence	1	1.49	0.230	0.03
**FA (Right DTC tract)**				
Group	1	19.69	**< 0.001**	0.32
Sequence	1	1.06	0.309	0.03
**MD (Left DTC tract)**				
Group	1	54.65	**< 0.001**	0.57
Sequence	1	86.63	**< 0.001**	0.68
**MD (Right DTC tract)**				
Group	1	40.12	**< 0.001**	0.49
Sequence	1	53.52	**< 0.001**	0.57
**RD (Left DTC tract)**				
Group	1	68.48	**< 0.001**	0.63
Sequence	1	48.68	**< 0.001**	0.54
**RD (Right DTC tract)**				
Group	1	59.84	**< 0.001**	0.59
Sequence	1	32.61	**< 0.001**	0.44

Note: Bold values indicate statistically significant results ($p < 0.05$).

Abbreviations: ANOVA, Analysis of variance; DTC tract, dentato-thalamo-cortical tract; Df, degrees of freedom; η2, Eta squared; FA, fractional anisotropy; MD, mean diffusivity; RD, radial diffusivity.

TABLE S3. Comparison of significant correlations between original Pearson's correlation and partial correlation controlling for age and sex.

FA values along the DTC tract	Clinical score	Pearson's correlation (original)	Partial correlation (corrected for age and sex)
Left DN→Left SCP	SARA	r=-0.44, *p*=0.040	r=-0.38, *p*=0.094
Left SCP	SARA	r=-0.55, *p*=0.008	r=-0.59, *p*=0.006
Left SCP→Right RN→Right Tha	SARA	r=-0.60, *p*=0.003	r=-0.61, *p*=0.004
Right SCP	SARA	r=-0.54, *p*=0.010	r=-0.56, *p*=0.010
Right SCP→Left RN→Left Tha	SARA	r=-0.50, *p*=0.018	r=-0.55, *p*=0.013
Left SCP→Right RN→Right Tha	ADL	r=-0.59, *p*=0.004	r=-0.60, *p*=0.005
Right SCP→Left RN→Left Tha	ADL	r=-0.55, *p*=0.009	r=-0.55, *p*=0.004

Note: p-values presented are uncorrected for multiple comparisons.

Abbreviations: FA, fractional anisotropy; DTC, dentato-thalamo-cortical; SARA, Scale for the Assessment and Rating of Ataxia; ADL, Activities of Daily Living; DN, dentate nucleus; SCP, superior cerebellar peduncles; RN, red nucleus; Tha, thalamus; r, correlation coefficient.

TABLE S4. Results of the Bayesian dynamic model within-group and between-group.

Parameter connectivity		Effect size	PP
**From**	**To**		
**Average parameter within-group**			
Left Dentate Nucleus	Left Dentate Nucleus	0.45	**1.00**
Left Dentate Nucleus	Right Thalamus	0.09	**0.83**
Right Thalamus	Left Dentate Nucleus	0.00	0.51
Right Thalamus	Right Thalamus	0.08	**0.81**
Right Thalamus	Right Motor Cortex	-0.41	**1.00**
Right Motor Cortex	Right Thalamus	0.05	0.74
Right Motor Cortex	Right Motor Cortex	0.30	**1.00**
Right Dentate Nucleus	Right Dentate Nucleus	0.62	**1.00**
Right Dentate Nucleus	Left Thalamus	0.17	**0.97**
Left Thalamus	Right Dentate Nucleus	-0.06	0.77
Left Thalamus	Left Thalamus	0.08	**0.80**
Left Thalamus	Left Motor Cortex	-0.36	**1.00**
Left Motor Cortex	Left Thalamus	0.06	0.77
Left Motor Cortex	Left Motor Cortex	0.26	**1.00**
**Difference parameter between FRDA and Control group**			
Left Dentate Nucleus	Left Dentate Nucleus	-0.00	0.50
Left Dentate Nucleus	Right Thalamus	-0.05	0.74
Right Thalamus	Left Dentate Nucleus	0.14	**0.95**
Right Thalamus	Right Thalamus	0.00	0.51
Right Thalamus	Right Motor Cortex	0.00	0.51
Right Motor Cortex	Right Thalamus	0.00	0.50

Right Motor Cortex	Right Motor Cortex	-0.00	0.51
Right Dentate Nucleus	Right Dentate Nucleus	-0.00	0.50
Right Dentate Nucleus	Left Thalamus	0.00	0.51
Left Thalamus	Right Dentate Nucleus	-0.00	0.51
Left Thalamus	Left Thalamus	-0.08	**0.82**
Left Thalamus	Left Motor Cortex	0.00	0.51
Left Motor Cortex	Left Thalamus	0.00	0.52
Left Motor Cortex	Left Motor Cortex	-0.00	0.52

Note: Bold value indicates clear evidence for a nonspurious effect (PP < 0.80).

Abbreviations: FRDA, Friedreich's ataxia; PP, posterior probability.

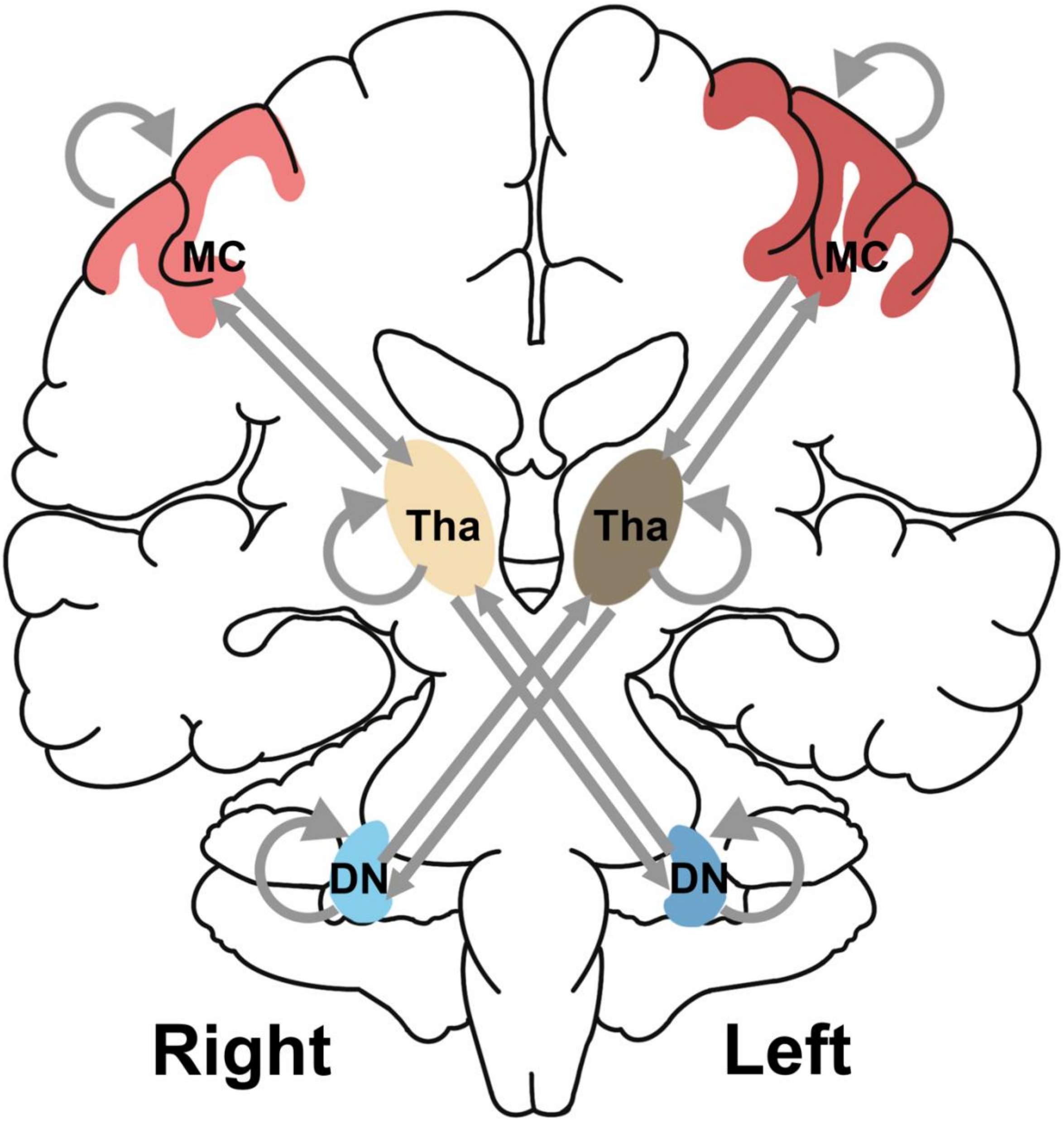

FIGURE S1. Illustration for a dynamic causal model network of the dentato-thalamo-cortical pathway. DN, dentate nucleus; Tha, thalamus; MC, motor cortex.

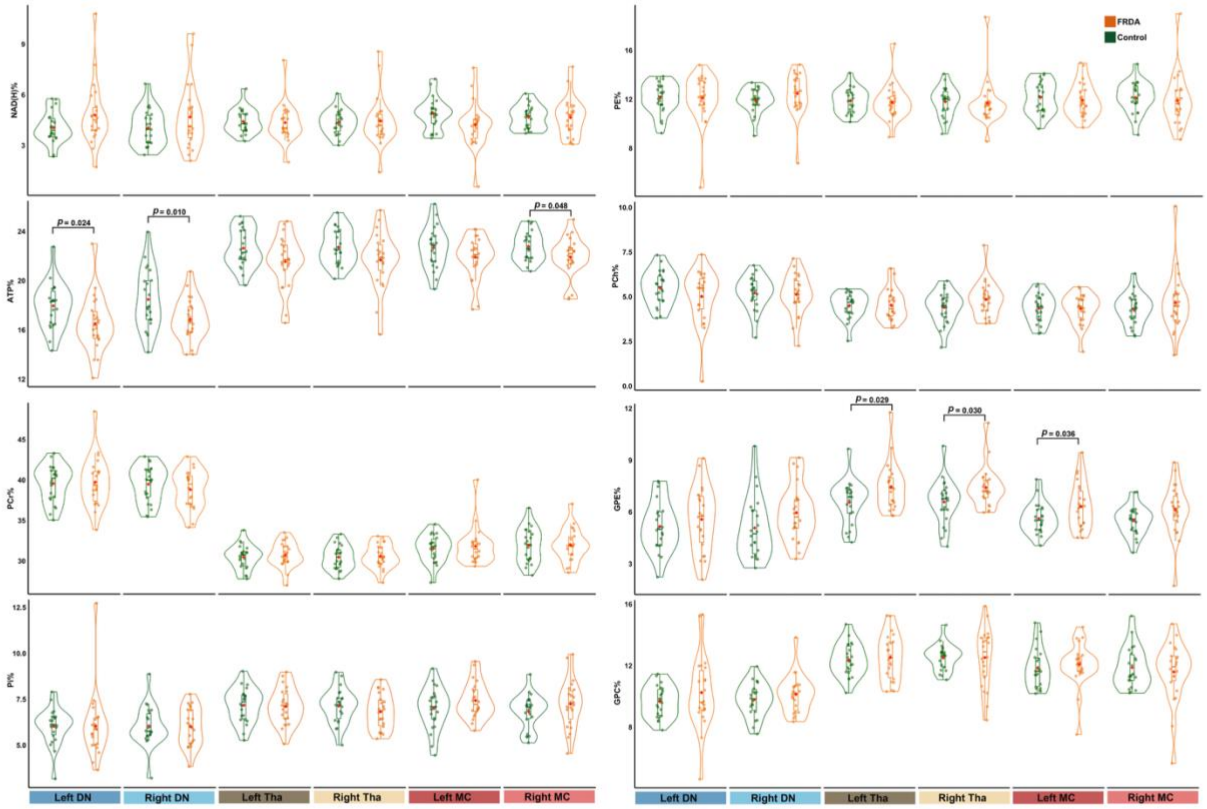

FIGURE S2. Group comparison of phosphate metabolites in each key brain region between patients with Friedreich's ataxia and controls. FRDA, Friedreich's ataxia; %, percentage of the total phosphorous signal; PCr, phosphocreatine; Pi, inorganic phosphate; ATP, adenosine triphosphate; NAD(H), nicotinamide adenine dinucleotide; PE, phosphoethanolamine; PCh, phosphocholine; GPE, glycerophosphoethanolamine; GPC, glycerophosphocholine; DN, dentate nucleus; Tha, thalamus; MC, motor cortex.

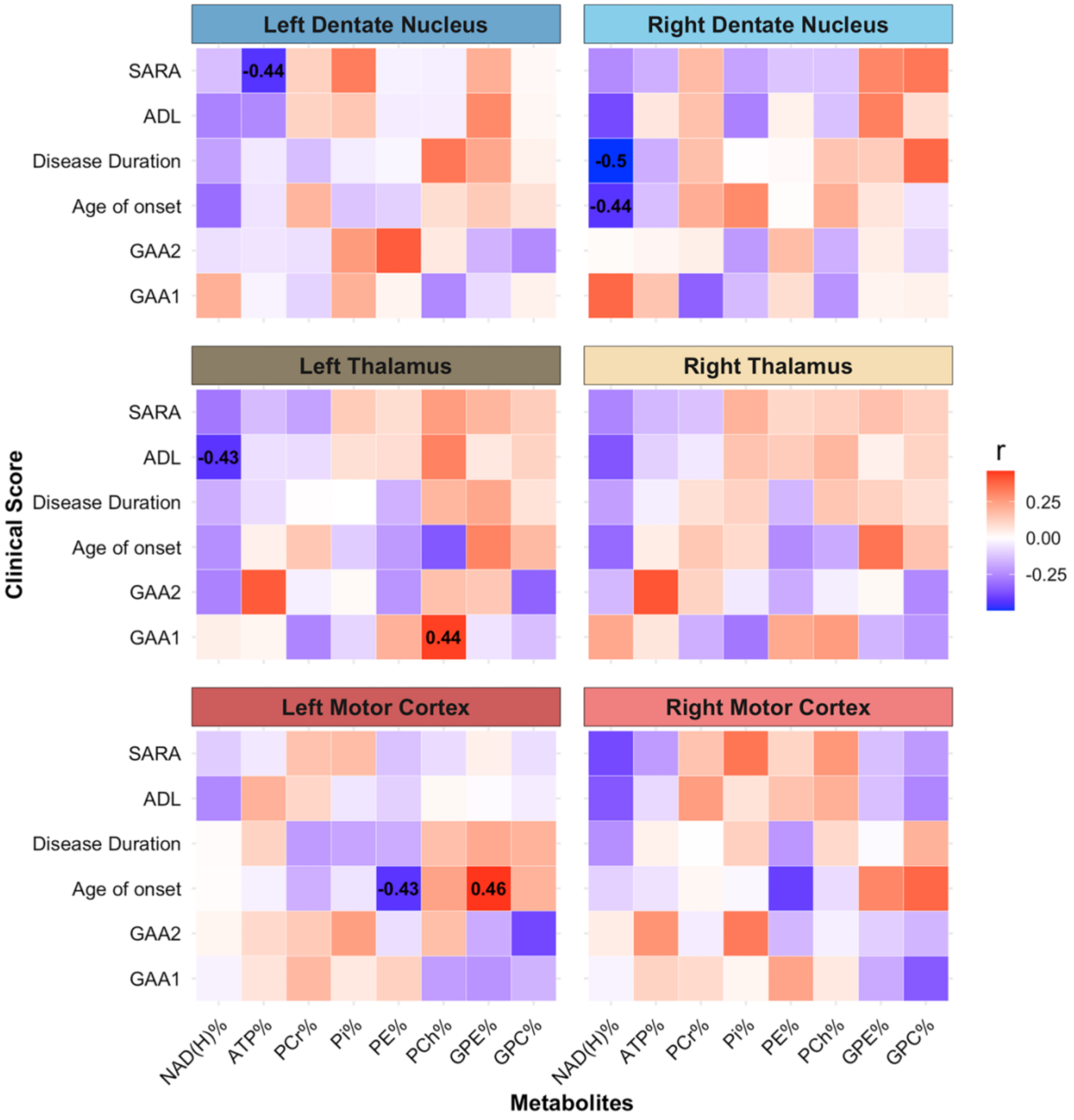

FIGURE S3. Correlation map illustrating Pearson's correlation coefficients between clinical scores and all metabolites in each key region along the dentato-thalamo-cortical pathway in Friedreich's ataxia patients. Only significant correlation coefficients ($p < 0.05$, uncorrected) are presented. GAA1, GAA repeat length on the smaller FXN allele; GAA2, GAA repeat length on the larger FXN allele; ADL, Activities of Daily Living; SARA, Scale for the Assessment and Rating of Ataxia; %, percentage of the total phosphorus signal; NAD(H), nicotinamide adenine dinucleotide; ATP, adenosine triphosphate; PCr, phosphocreatine; Pi,

inorganic phosphate; PE, phosphoethanolamine; PCh, phosphocholine; GPE, glycerophosphoethanolamine; GPC, glycerophosphocholine.

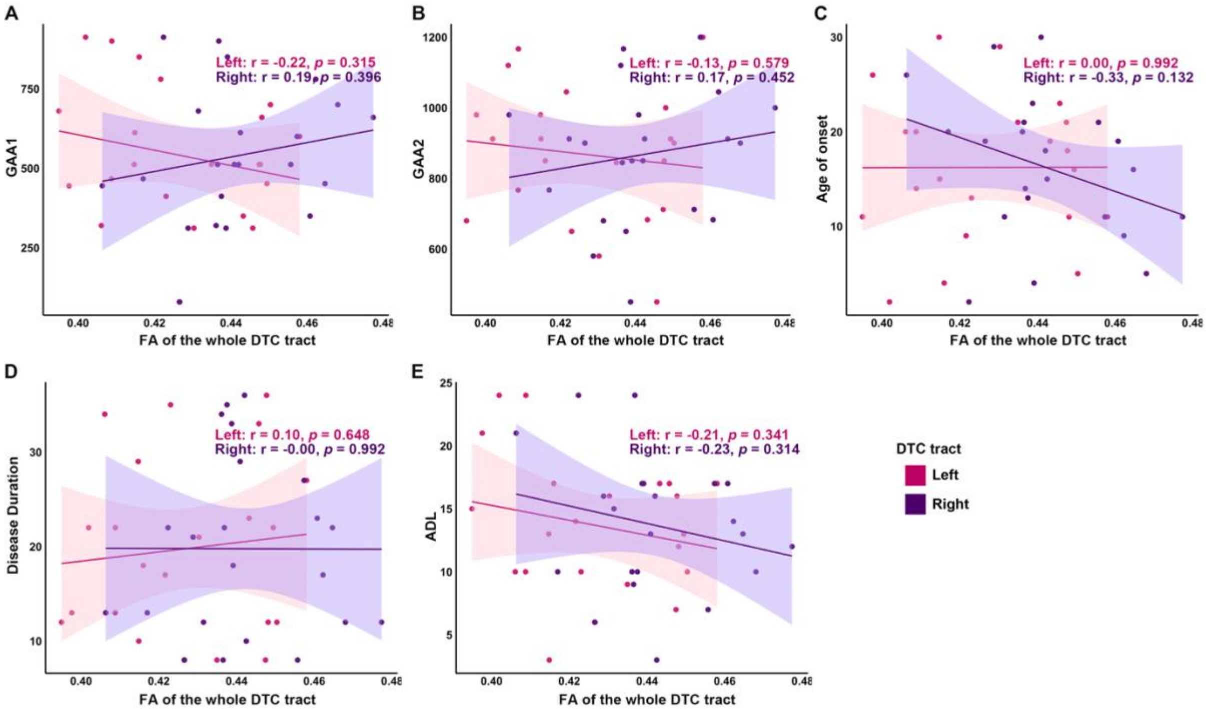

FIGURE S4. Scatterplots and linear regression plots between clinical scores and mean FA of the whole dentato-thalamo-cortical tract in Friedreich's ataxia patients. *p*-values presented are uncorrected for multiple comparisons. DTC, dentato-thalamo-cortical; FA, fractional anisotropy; GAA1, GAA repeat length on the smaller FXN allele; GAA2, GAA repeat length on the larger FXN allele; ADL, Activities of Daily Living; r, Pearson's correlation coefficient.

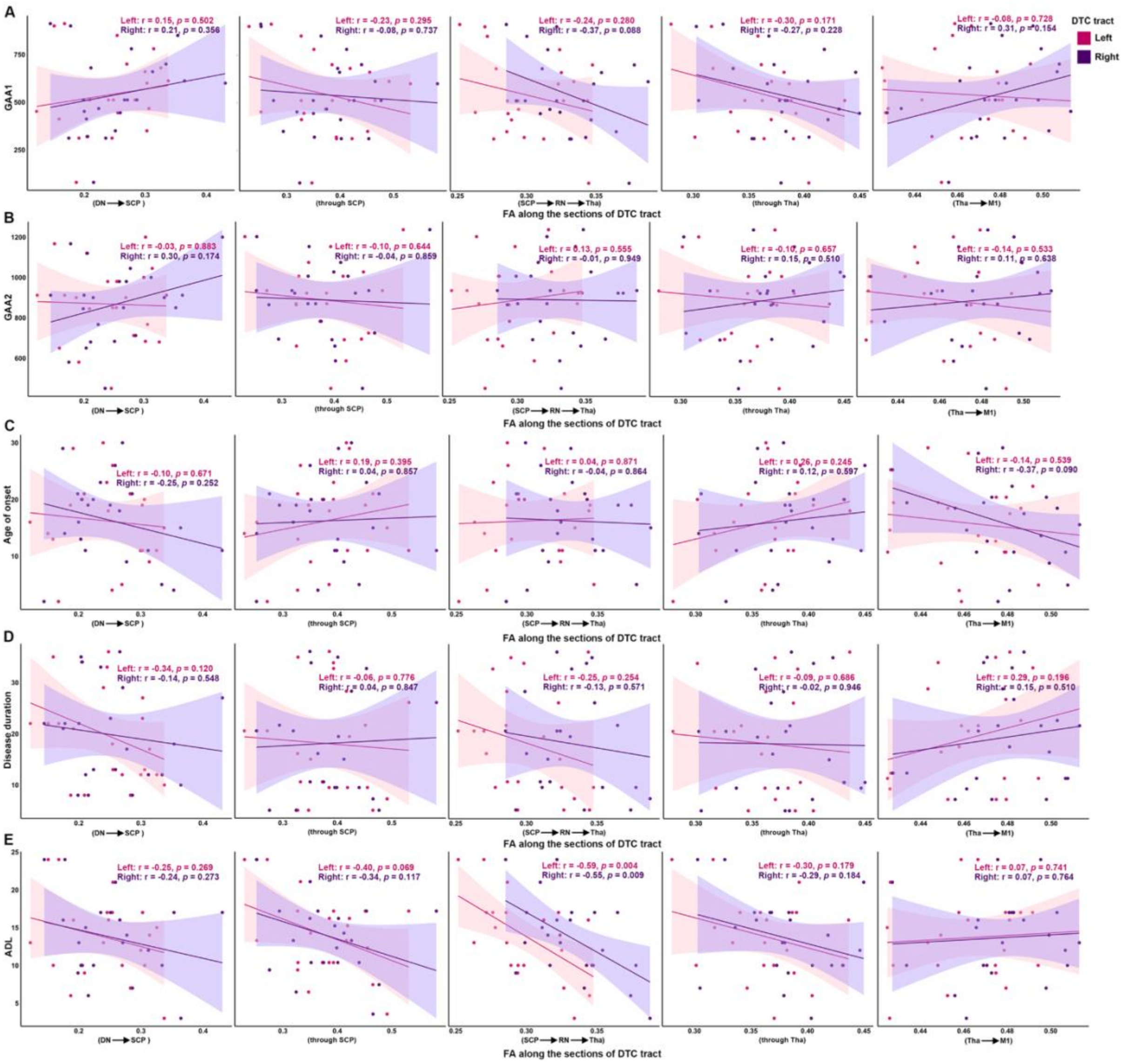

FIGURE S5. Scatterplots and linear regression plots between average FA along each section of the dentato-thalamo-cortical tract and clinical scores in Friedreich's ataxia patients. *p*-values presented are uncorrected for multiple comparisons. DTC, dentato-thalamo-cortical; FA, fractional anisotropy; GAA1, GAA repeat length on the smaller FXN allele; GAA2, GAA repeat length on the larger FXN allele; ADL, Activities of Daily Living; DN, dentate nucleus; SCP, superior cerebellar peduncles; RN, red nucleus; Tha, thalamus; MC, motor cortex; r, Pearson's correlation coefficient.

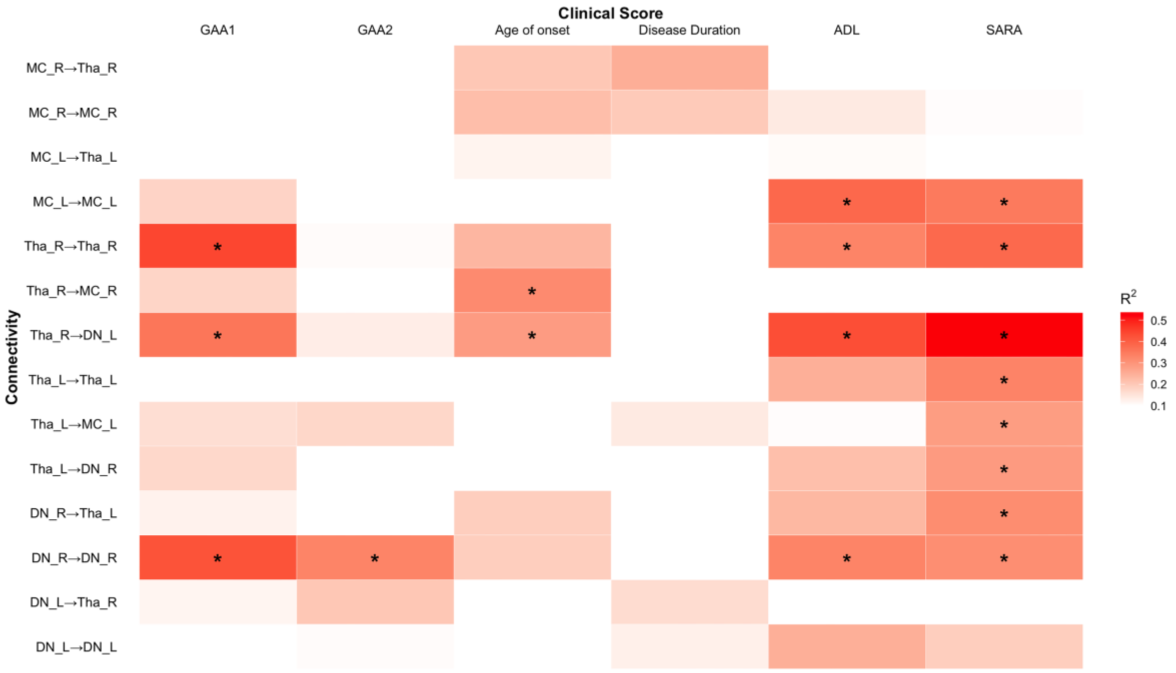

FIGURE S6. Quadratic regression analyses between effective connections and clinical scores in Friedreich's ataxia patients. Only models with significance (*$p < 0.05$, uncorrected) and $R^2 > 0.1$ are presented. GAA1, GAA repeat length on the smaller FXN allele; GAA2, GAA repeat length on the larger FXN allele; ADL, Activities of Daily Living; SARA, Scale for the Assessment and Rating of Ataxia; DN, dentate nucleus; Tha, thalamus; MC, motor cortex; R^2, coefficient of determination.

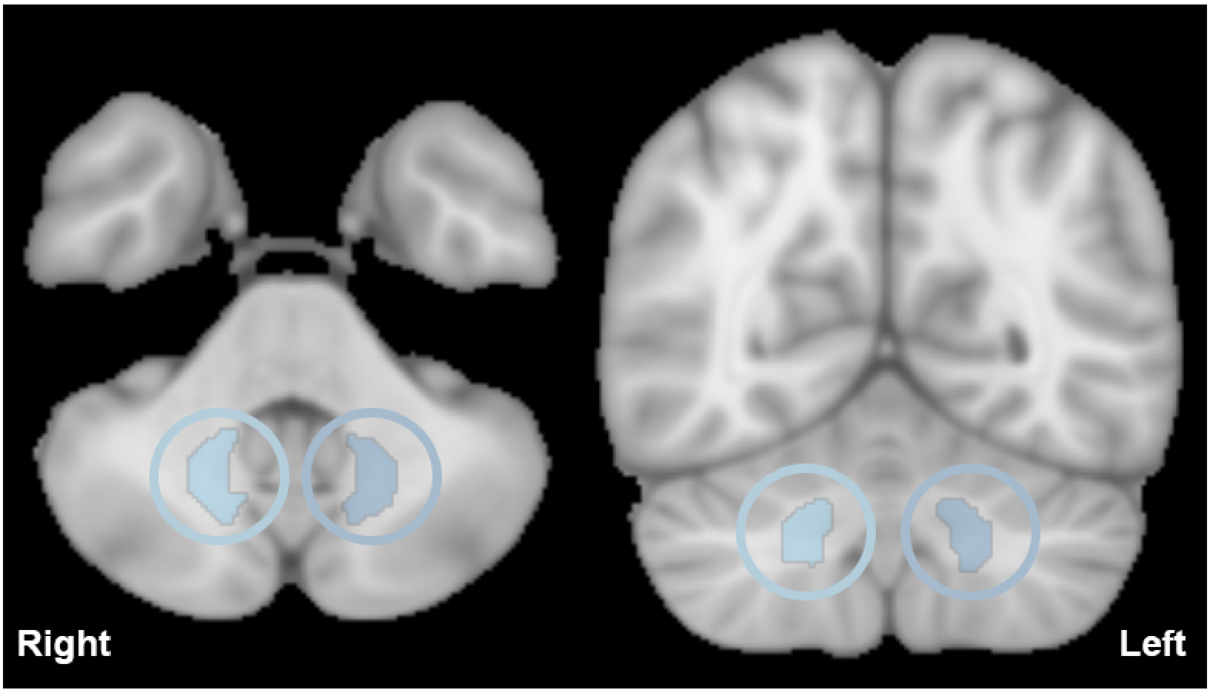

FIGURE S7. Dentate nucleus selection of ^{31}P-MRS displayed on axial and coronal anatomical images. Solid masks are the dentate nucleus ROIs used to extract the values; circles indicate an approximation of the effective spherical size.

Supplementary References

1. Pohmann R, von Kienlin M. Accurate phosphorus metabolite images of the human heart by 3D acquisition-weighted CSI. Magn Reson Med 2001;45(5):817-826.

2. Jia XZ, Wang J, Sun HY, et al. RESTplus: an improved toolkit for resting-state functional magnetic resonance imaging data processing. Sci Bull (Beijing) 2019;64(14):953-954.

3. Wilson M, Reynolds G, Kauppinen RA, Arvanitis TN, Peet AC. A constrained least-squares approach to the automated quantitation of in vivo (1)H magnetic resonance spectroscopy data. Magn Reson Med 2011;65(1):1-12.

4. De Graaf RA. In vivo NMR spectroscopy: principles and techniques: John Wiley & Sons, 2019.

5. Wilson M, Andronesi O, Barker PB, et al. Methodological consensus on clinical proton MRS of the brain: Review and recommendations. Magn Reson Med 2019;82(2):527-550.

6. Avants BB, Tustison NJ, Song G, Cook PA, Klein A, Gee JC. A reproducible evaluation of ANTs similarity metric performance in brain image registration. Neuroimage 2011;54(3):2033-2044.

7. Avants BB, Tustison NJ, Stauffer M, Song G, Wu B, Gee JC. The Insight ToolKit image registration framework. Front Neuroinform 2014;8:44.

8. Henschel L, Conjeti S, Estrada S, Diers K, Fischl B, Reuter M. FastSurfer - A fast and accurate deep learning based neuroimaging pipeline. Neuroimage 2020;219:117012.

9. Avants BB, Epstein CL, Grossman M, Gee JC. Symmetric diffeomorphic image registration with cross-correlation: evaluating automated labeling of elderly and neurodegenerative brain. Med Image Anal 2008;12(1):26-41.

10. Tournier JD, Smith R, Raffelt D, et al. MRtrix3: A fast, flexible and open software framework for medical image processing and visualisation. Neuroimage 2019;202:116137.

11. Jeurissen B, Tournier JD, Dhollander T, Connelly A, Sijbers J. Multi-tissue constrained spherical deconvolution for improved analysis of multi-shell diffusion MRI data. Neuroimage 2014;103:411-426.

12. Tournier JD, Calamante F, Connelly A. Robust determination of the fibre orientation distribution in diffusion MRI: non-negativity constrained super-resolved spherical deconvolution. Neuroimage 2007;35(4):1459-1472.
